# Sonographic and Elastographic Features of Extra- and Intrathyroidal Ectopic Thymus Mimicking Malignancy: Differential Diagnosis in Children

**DOI:** 10.3389/fendo.2019.00223

**Published:** 2019-04-10

**Authors:** Magdalena Stasiak, Zbigniew Adamczewski, Renata Stawerska, Tomasz Krawczyk, Monika Tomaszewska, Andrzej Lewiński

**Affiliations:** ^1^Department of Endocrinology and Metabolic Diseases, Polish Mother's Memorial Hospital-Research Institute, Lodz, Poland; ^2^Department of Endocrinology and Metabolic Diseases, Medical University of Lodz, Lodz, Poland; ^3^Department of Pathology, Polish Mother's Memorial Hospital-Research Institute, Lodz, Poland; ^4^Department of Pediatrics, Oncology, Hematology and Diabetology, Central Teaching Hospital of the Medical University of Lodz, Lodz, Poland

**Keywords:** ectopic thymus, thyroid, elastography, ultrasound, thyroid cancer, metastatic lymph node

## Abstract

Thyroid nodules with ultrasound (US) cancer risk features and extra-thyroid lesions suggesting malignant lymph nodes, require prompt diagnosis, especially in children. The US pattern of intrathyroidal ectopic thymus (IET) can strongly suggest papillary thyroid carcinoma (PTC). The extra-thyroid ectopic thymic tissue (EET) can mimic pathological lymph nodes in US. The aim of the study has been to demonstrate US features and diagnostic methods, allowing finally to confirm the presence of IET and EET in children. The US and elastographic features of 16 ectopic thymic tissue (ET) lesions were analyzed so as to describe the typical characteristics of ET and to define the best method to differentiate ET and malignant lesions. Among 16 analyzed lesions, 11 lesions were IET, and 5 were EET adjacent to the thyroid connective tissue capsule. Most of IET were located in the middle part of the right lobe and were fusiform or oval in shape. All the lesions were solid, hypoechoic, and heterogeneous with bright internal echoes. Among IET, 73% of lesions had well- or very well-defined margins. In strain elastography of IET lesions, the strain ratio was similar in all lesions, and its value ranged from 0.95 to 1.09. Despite the low prevalence of IET and cervical EET, clinicians and radiologists should be aware of US characteristics of such lesions. The confirmation of their benign character is absolutely required. Elastography is a useful tool to initially differentiate PTC and IET. However, due to high risk of malignancy in thyroid lesions in children, similarity of US features of PTC and IET, and due to the possibility of malignancy in ET, only cytological evaluation provides definitive diagnosis.

## Introduction

Ultrasonography (US) is an accurate non-invasive diagnostic method commonly used in neck imaging. This is the first line tool in the diagnosis of thyroid gland nodules. In children, a frequent reason for neck US is the diagnosis of enlarged lymph nodes or other palpable neck nodules, but US is sometimes performed also due to family history of thyroid diseases or as a part of population studies. The increasing availability of US examination results in accidental findings of many more thyroid lesions than there used to be. Estimates from US and postmortem examinations suggest that 1–1.5% of children and up to 13% of older adolescents or young adults have thyroid nodules (1). The risk of cancer in thyroid nodules in children is 22–26% and is much higher than in adults (up to 5–10%) (1). Thus, in accordance with current guidelines, any thyroid lesion found in a child, except for pure cysts, requires thorough diagnosis including fine needle aspiration biopsy (FNAB). The size of the thyroid nodule cannot be a qualification criterion for FNAB because the child's body and thyroid are smaller than an adult's. Therefore, even small but suspicious nodules should undergo FNAB. Bilateral occurrence of focal lesions in a child does not reduce diagnostic alertness because thyroid cancer in children is often multifocal and bilateral (1, 2). In children, disease progression at the time of diagnosis is usually significant, with the presence of lymph node metastases in most cases and—in over 20% of cases—also distant lung metastases (1, 2). Hence, careful US examination of lymph nodes is required in every child with thyroid lesions. Sonographic features suggesting malignant thyroid lesion include solid and hypoechoic tumor pattern, irregular tumor margins, tumor shape (taller than wide), presence of microcalcifications, and increased tumor vascularization. Abnormal, hypoechogenic structure within the neck, containing microcalcifications, and not showing central vascularization may correspond to a pathological neoplastic lymph node.

A thymus is a lymphatic organ involved in the differentiation of T lymphocytes. During embryogenesis, the thymus is formed from the ectoderm of the third branchial cleft and the endoderm of the third branchial pouch. The definitive thymus is formed by fusion of the right and left thymic primordials. Then it descends to the upper anterior mediastinum (3, 4). Aberrant thymic migration may lead to ectopic thymus location, including intrathyroidal locus. The prevalence of ectopic neck thymic tissue (ET) in children is very low and was reported as 0.99% (5) to 1.8% (6).

The US pattern of intrathyroidal ectopic thymus (IET) can strongly suggest papillary thyroid carcinoma (PTC), which is the most common thyroid cancer in children. The IET is usually a hypoechogenic lesion, with pronounced numerous punctate or linear bright internal echoes that suggest microcalcifications. In addition, the lesion margins may be irregular. Such features suggest PTC and require precise differential diagnosis. The extra-thyroid ectopic thymic tissue (EET) is most often located in the vicinity of the thyroid gland, usually close to the lower pole of the one of the lobes. For an inexperienced ultrasonographer, the US image of such a structure may suggest the presence of a pathological lymph node.

Strain elastography is a method which evaluates tissue stiffness (elasticity) by measuring the degree of tissue deformation in response to mechanical compression (7, 8). In this method, the stiffness of the thyroid lesion is compared to the adjacent healthy thyroid tissue and the difference in relative stiffness is presented as the strain ratio (SR). A SR value close to 1.0 means a similar relative stiffness for both evaluated tissues, and the higher the SR, the more suspicious the examined lesion is.

The aim of the study has been to present US features and diagnostic methods that will finally allow for confirmation of the presence of ET in children who were referred to our center in order to definitively diagnose suspicious thyroid nodules. Most of the presented children had already been scheduled for thyroid surgery in other centers.

## Materials and Methods

Medical data of nine children with 16 lesions, who were referred to the Department of Endocrinology and Metabolic Diseases, Polish Mothers' Memorial Hospital–Research Institute, Lodz, Poland, with suspected PTC or suspicion of neoplastic lymph node were analyzed. In 11 of the cases, the US image of thyroid gland required differentiation with IET and in the remaining 5, the EET mimicked metastatic lymph nodes. After admission, patients had laboratory tests performed, including thyrotropin (TSH), free triiodothyronine (FT3), free thyroxine (FT4), parathyroid hormone (PTH) levels, anti-thyroglobulin antibodies (aTg), anti-thyroid peroxidase antibodies (aTPO), and TSH receptor antibodies (TRAb). All parameters were measured by electrochemiluminescence immunoassay (ECLIA) with a Cobas e601 analyzer (Roche Diagnostics, USA). Ultrasound examination was performed in every patient using a 7–14 MHz linear transducer (AplioXG, Toshiba Medical Systems Corp., Shimoishigami, Otawara-shi, Tochigi-ken, Japan). Scanning was performed in supine position with a pad under the patients' shoulders to provide optimum neck extension. Strain elastography was performed in all IET lesions (AplioXG, Toshiba Medical Systems Corp., Shimoishigami, Otawara-shi, Tochigi-ken, Japan).

FNAB with US guidance was performed in all patients using a 23-gauge needle. In all patients FNAB procedures were performed under moderate sedation or general anesthesia. All cytological evaluations were carried out by the same pathologist, who had more than 20 years of experience. Smears were cytologically evaluated and the presence of small lymphocytes with scattered epithelioid cells, without the presence of macrophages, histiocytes, or other cell types (e.g., eosinophils and plasma cells) was considered as a result typical for thymic tissue. The absence of lymphocytes of different stages of differentiation together with the absence of macrophages and other cells typically occurring in lymph nodes were required for differentiation with lymph nodes or other lymphatic tissues. The absence of oncotic follicular cells and plasma cells allowed us to cytologically exclude lymphocytic thyroiditis.

In all cases, written informed consent for all performed procedures was obtained from the patients' parents.

## Results

### Clinical Features

The mean age of our patients was 5.8 years, ranging from 6 months to 11 years. The male to female ratio was 3:1. None of the children had any family history of thyroid cancer nor did they have medical history of irradiation. We did not measure calcitonin levels in the evaluated patients, as routine calcitonin assessment in diagnostics of thyroid nodules in children not harboring germline *RET* proto-oncogene mutation is not recommended (2). However, in 4 children the level of calcitonin was assessed in other centers and in all of them it was lower than 10 pg/ml, ranging from 4 to 7 pg/ml. In all patients in whom thyroid hormone tests were performed, all the results were normal for the patients' age. Anti-thyroid antibodies were negative in all cases. Clinical characteristics of the patients are presented in Supplementary Table 1.

### Ultrasonographic Features

A total of 16 ectopic thymic tissues were found in 9 patients. Eleven lesions were IET and five were EET adjacent to the thyroid capsule. Ultrasound features of the studied lesions are presented in Tables 1, 2. IET size varied from 4 to 14 mm with the mean largest dimension at 6.5 mm, while EET size ranged from 7 to 18 mm with a mean largest dimension of 14 mm. Among IET, 7 lesions were located in right lobe and 4 in left lobe, 7 were found in the middle part of thyroid lobe, 4 in the lower part, and none in the upper part of the thyroid lobe. In three children, IET was located bilaterally, while in four children IET was unilateral. Extrathyroidal lesions were unilateral in 3 children, and all of them were located directly below the right lobe. In one child EET was bilateral. Coexistence of EET and IET was found in 2 children (Table 1).

**Table 1:** Ultrasound characteristics of the patients with ectopic thymic tissue.

**Case**	**Age**	**Gender**	**No of lesions in one child**	**Location**	**Size (mm)**	**Bilateral**
1	6 mo	F	1	EET below RL	12 × 8 × 18	No
2	5 yr	M	2	RL inferior part	RL: 5 × 4 × 5	Yes
				LL middle part	LL: 5 × 2 × 5	
3	7 yr	M	3	RL middle part	RL: 5 × 2 × 6	Yes
				LL middle part	LL: 11 × 2 × 12	
				EET below the RL	Below RL: 10 × 5 × 12	
4	4 yr	M	2	RL middle part	RL: 4 × 2 × 6	No
				EET below RL	Below RL: 5 × 4 × 7	
5	10 yr	F	1	RL middle part	6 × 2 × 7	No
6	4 yr	M	3	RL inferior part	RL inferior: 3 × 2 × 4	Yes
				RL middle part	RL middle: 7 × 3 × 6	
				LL inferior part	LL: 5 × 6 × 11	
7	5 yr	F	1	LL inferior part	7 × 6 × 14	No
8	11 yr	M	2	EET below RL	Below RL: 9 × 9 × 17	Yes
				EET below LL	Below LL: 7 × 13 × 16	
9	6 yr	M	1	RL middle part	6 × 4 × 7	No

*EET, extrathyroidal ectopic thymus; F, female; IET, intrathyroidal ectopic thymus; LL, left lobe; M, male; mo, months; RL, right lobe; yr, years*.

**Table 2:** Ultrasound features and strain ratio (SR) of lesions diagnosed as ectopic thymic tissue.

**No**	**Size (mm)**	**Shape**	**Internal echoes**	**Margins**	**Vascularity**	**Elastography SR**	**Cytology**
1	5 × 4 × 5	Heart	A few linear bright echoes in the middle, hypoechoic margin	Well-defined	low	0.97	Typical for thymus
2	5 × 2 × 5	Fusiform	A few linear bright echoes in the middle, hypoechoic margin	Very well-defined	low	1.09	Typical for thymus
3	5 × 2 × 6	Fusiform	Numerous punctual and linear bright internal echoes scattered unevenly	Blurred	low	1.06	Typical for thymus
4	11 × 2 × 12	Longitudinal	Numerous punctual and linear bright internal echoes scattered mainly in the medial part	Very well-defined	low	0.95	Typical for thymus
5	4 × 2 × 6	Fusiform	Several punctual and linear bright internal echoes scattered unevenly	Well-defined	low	0.96	Typical for thymus
6	6 × 2 × 7	Oval	Three linear bright internal echoes in the middle, hypoechoic margin	Well-defined	no	1.06	Typical for thymus
7	3 × 2 × 4	Oval	Very few linear bright internal echoes in the middle, hypoechoic margin	Blurred	no	0.99	Typical for thymus
8	7 × 3 × 6	Fusiform	A few linear bright internal echoes in the middle, hypoechoic margin	Well-defined	low	1.09	Typical for thymus
9	5 × 6 × 11	Oval	A few linear bright internal echoes scattered mainly in the middle, hypoechoic margin	Well-defined	low	1.01	Typical for thymus
10	7 × 6 × 14	Oval	Several punctual and linear bright internal echoes scattered unevenly	Well-defined	low	0.97	Typical for thymus
11	6 × 4 × 7	Oval	A few scattered linear bright internal echoes	Blurred	low	1.03	Typical for thymus
12	12 × 8 × 18	Oval	A few linear bright echoes scattered unevenly	Well-defined	low	NA	Typical for thymus
13	7 × 4 × 5	Triangular	A few punctual and linear bright internal echoes scattered unevenly	Very well-defined	low	NA	Typical for thymus
14	10 × 5 × 12	Triangular	Several punctual and linear bright internal echoes scattered unevenly	Very well-defined	low	NA	Typical for thymus
15	9 × 9 × 17	Oval	Several linear bright internal echoes scattered quite unevenly	Very well-defined	low	NA	Typical for thymus
16	7 × 13 × 16	Oval	Several linear bright internal echoes scattered quite evenly	Very well-defined	low	NA	Typical for thymus

*Lesions are presented according to the location: first 11 lesions are IET, second 5 lesions are EET*.

*EET, extrathyroidal ectopic thymus; LL, left lobe; NA, not applicable; RL, right lobe; SR, strain ratio*.

**Figure 1:**
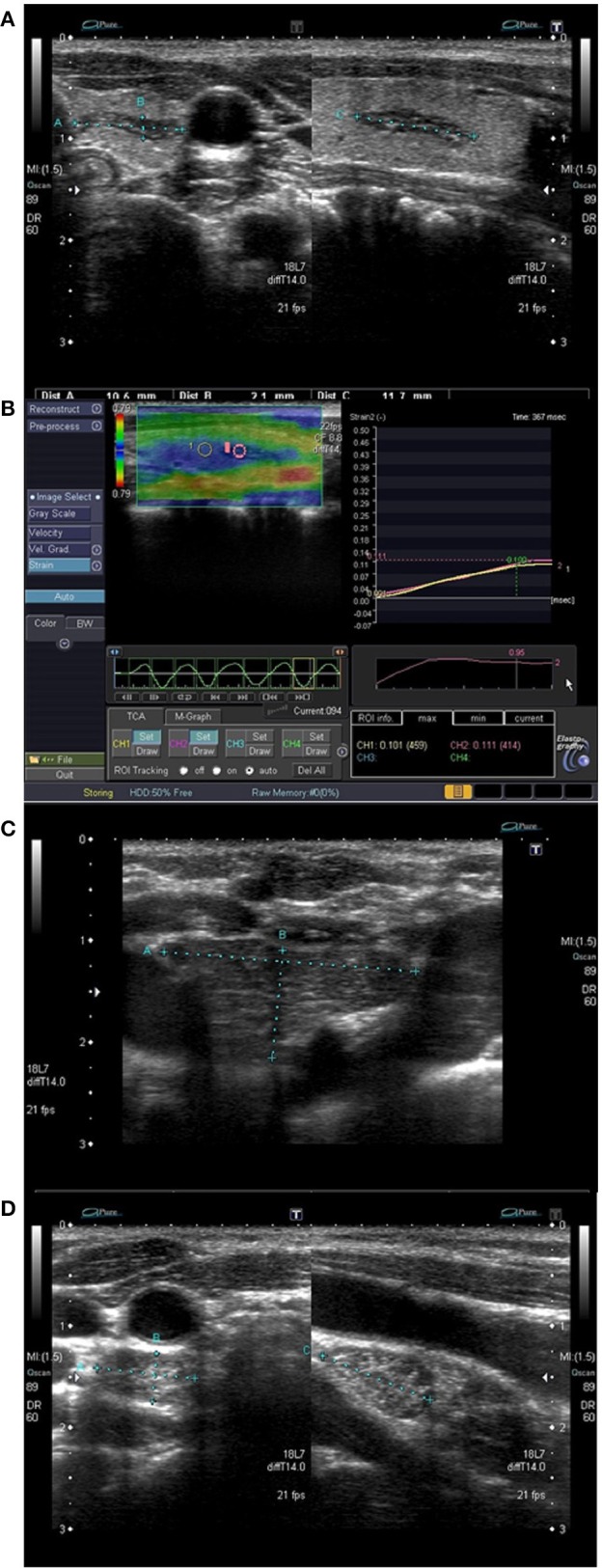
Patient No. 3 (Table 1). **(A)** A longitudinal intrathyroid ectopic thymus (IET) of the left thyroid lobe; **(B)** Elastography of the IET; **(C)** Normal thymus in physiologic location; **(D)** Extrathyroid ectopic thymus below the right thyroid lobe.

**Figure 2:**
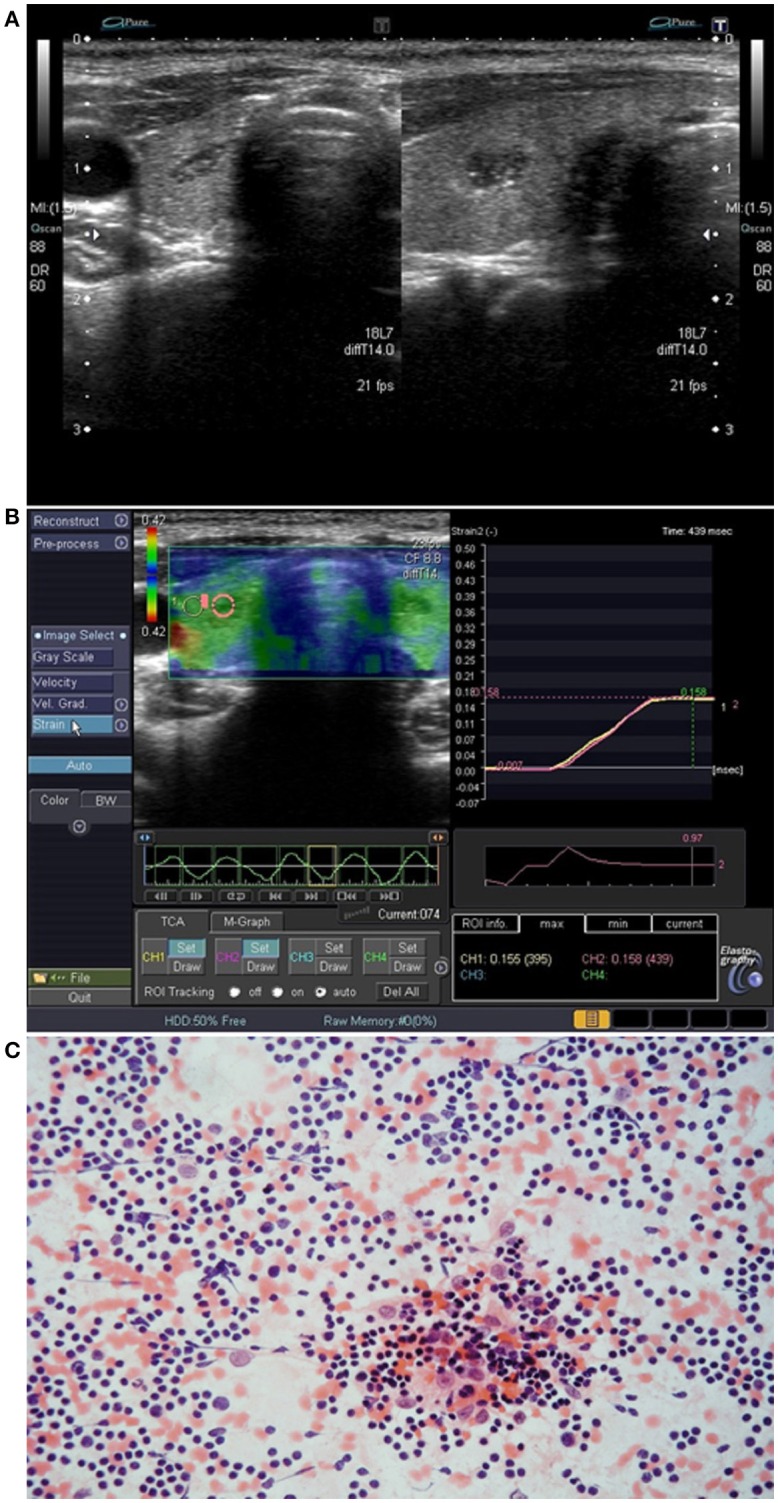
Patient No. 2 (Table 1). **(A)** Intrathyroid ectopic thymus (IET) with well-defined margins in the right thyroid lobe; **(B)** Elastography of the IET; **(C)** Cytological smear shows numerous small lymphocytes with scattered epithelioid cells (hematoxylin-eosin staining; light microscopy, magnification × 80).

**Figure 3:**
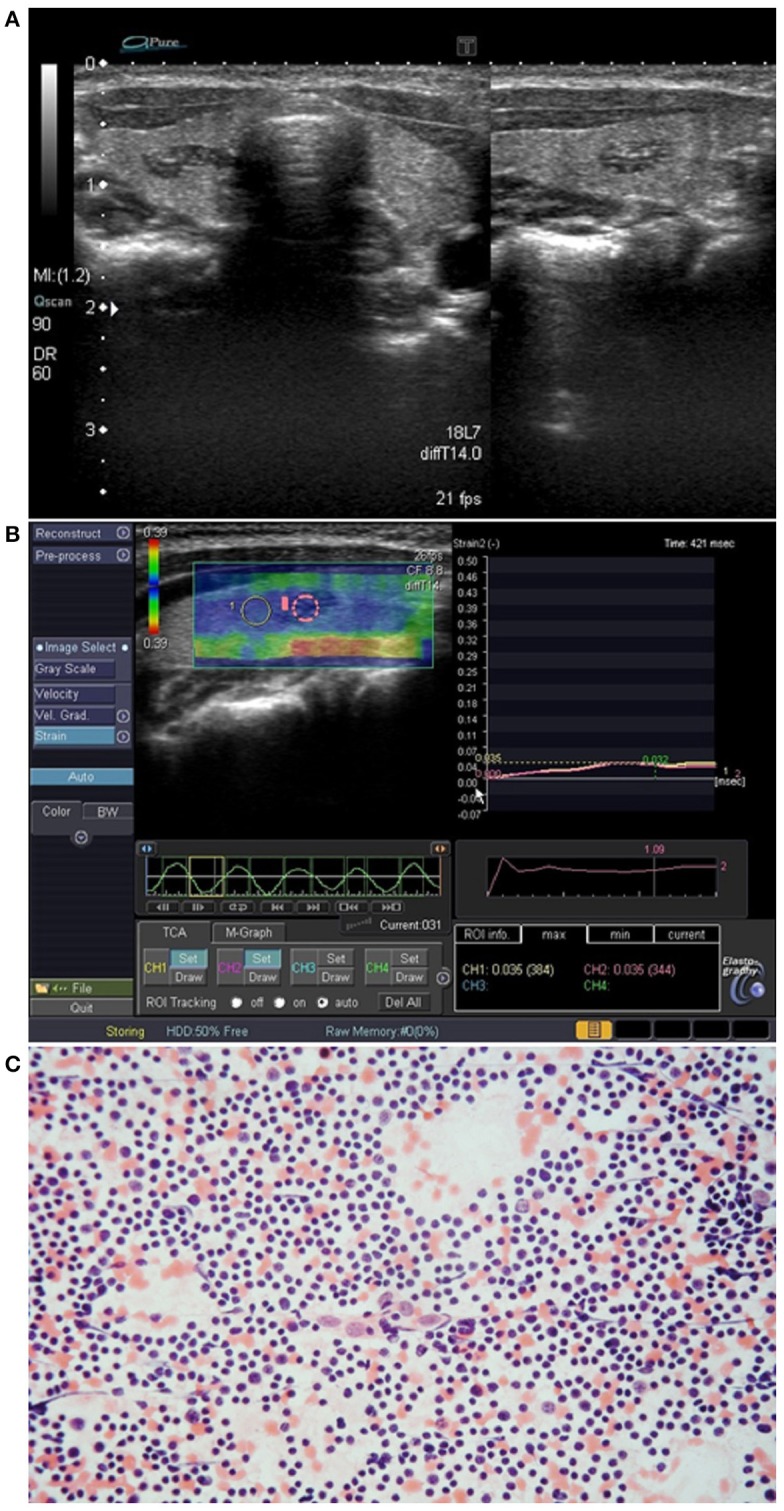
Patient No. 6 (Table 1). **(A)** Intrathyroid ectopic thymus (IET) in the right thyroid lobe; **(B)** Elastography of the IET; **(C)** Cytological smear shows numerous small lymphocytes with scattered epithelioid cells (hematoxylin-eosin staining; light microscopy, magnification × 80).

**Figure 4:**
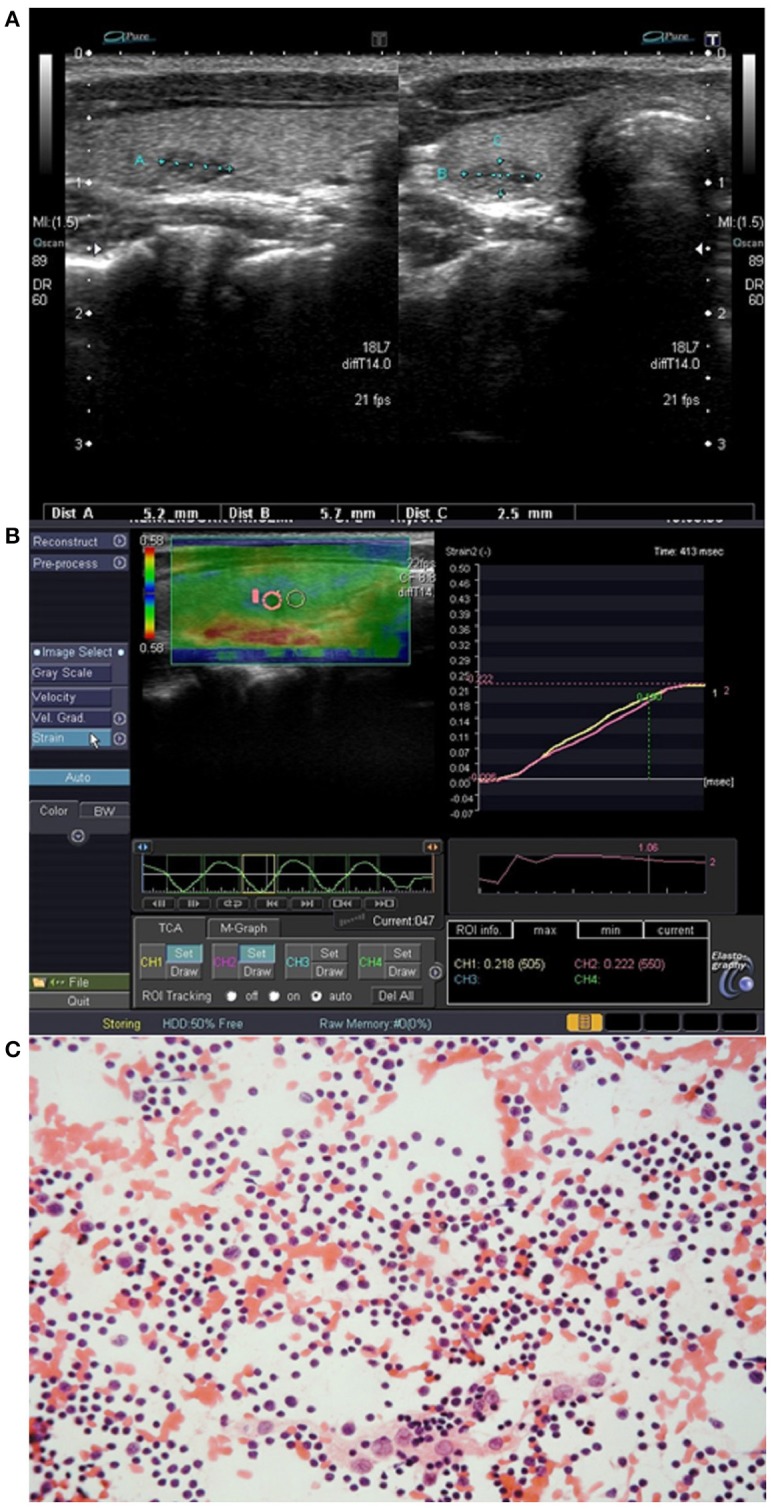
Patient No. 3 (Table 1). **(A)** Intrathyroid ectopic thymus (IET) with blurred margins in the right thyroid lobe; **(B)** Elastography of the IET; **(C)** Cytological smear shows numerous small lymphocytes with scattered epithelioid cells (hematoxylin-eosin staining; light microscopy, magnification × 80).

Most of the cases of IET were fusiform (4 lesions) (Figure 3A) or oval (5 lesions) (Figure 4A) in shape, while extrathyroidal thymic tissue lesions were triangular (2 lesion) (Figure 1D), or oval (3 lesion). Among IET cases, there was one lesion of non-typical heart shape and one lesion of very characteristic longitudinal shape (Figure 1A), which strongly suggested ectopic tissue. In US, all the lesions were solid, hypoechoic, and heterogeneous with bright internal echoes (Figures 1–4). In all lesions, linear bright echoes were present, while punctual echoes were observed in 6 lesions only. The number of bright echoes varied, from numerous to only a few (3–4) echoes. In 6 lesions, the bright echoes were located in the middle of the lesion, with a hypoechoic margin (Figure 1A), while in the remaining 9 lesions they were scattered, in 7 cases unevenly (Figures 2A, 3A). Among IET cases, 73% (8/11) had well or very well-defined margins and only 3 lesions had blurred margins (27%) (Figure 4A). All extrathyroidal lesions had well-defined margins (Figure 1D). In 6 of the IET nodules, blood flow in power Doppler (PD) evaluation was decreased, and in the remaining 5 IET cases no blood flow was observed (Table 2). All extrathyroidal lesions had decreased blood flow in PD (Table 2). In all cases, the EET resembled an US appearance of a normal thymus, which was present in a normal location in every patient (Figure 1C).

### Elastographic Features

In strain elastography of IET lesions, the SR was similar in all lesions, and its value ranged from 0.95 to 1.09, mean 1.02 (Table 2, Figures 1B, 2B, 3B, 4B), thus the stiffness of IET was comparable to adjacent thyroid tissue.

### Cytomorphological Features

The cytology findings included lymphoid cells that were usually numerous, always with a predominance of small lymphocytes with scattered epithelioid cells (Figures 2C, 3C, 4C). None of the smears included thyroid follicular cells, macrophages, histiocytes or other cell types (e.g., eosinophils and plasma cells). The absence of lymphocytes of different stages of differentiation, macrophages and other cells typically occurring in lymph nodes allowed for differentiation with lymph nodes or other lymphatic tissues. The absence of thyroid follicular cells, oncotic cells, histiocytes, macrophages and plasma cells allowed us to cytologically exclude autoimmune thyroiditis. In all cases, the material was sufficient for cytological analysis and a cytological confirmation of ET was possible.

## Discussion

In children, finding of a pathological lesion in the thyroid or in another cervical location always arouses diagnostic alertness. Many extrathyroidal lesions on a child's neck are reactive lymph nodes that only require observation. However, thyroid nodules other than pure cysts and every cervical mass with suspicious US features require further accurate diagnosis to exclude malignancy.

There are a few reports on the prevalence and ultrasound characteristics of IET, which were carried out on large groups of patients, but the presence of IET was confirmed in them only on the basis of US imagery without cytological evaluation (4, 5). In many reports on the US characteristics of ET, cytological evaluation was not performed although the number of analyzed lesions was similar or even lower than ours (3, 9, 10). This approach involves the potentially high risk of misdiagnosis of children with suspicious thyroid lesions, some of which can be malignant. Reports in which IET suspicion was confirmed cytologically include small groups of patients with a dozen or so (2–15 subjects) IET lesions analyzed (6, 11–13). The group presented in this study is therefore one of the largest with cytological IET confirmation described so far. To the best of our knowledge, this is the first study in which elastography was used in the diagnosis of IET, and the first study that demonstrated the usefulness of this method in the initial evaluation of IET-like lesions. We have shown that the stiffness of IET tissue is similar to the stiffness of the surrounding healthy thyroid tissue (mean SR 1.02). This is of great importance in the differentiation of IET and PTC, since PTC is known to be significantly stiffer than thyroid tissue (14). Follicular cancer, which is often soft in elastography and different in US pattern, is extremely rare in children. The usefulness of elastography is therefore even greater, because it can be assumed that a US IET-like lesion with stiffness similar to the thyroid tissue is actually IET. If the stiffness of the lesion is greater than the surrounding thyroid tissue, we should suspect PTC, even if the US pattern of the lesion resembles IET.

The US differential diagnosis between IET and suspicious thyroid nodules is challenging. Children with hypoechoic nodules with microcalcification-like echoes are often referred for surgery due to strong suspicion of malignancy.

In our study, the analyzed IET cases were located mostly in the middle part of the thyroid lobe (7 lesions) and less frequently in the lower part (4 lesions). In nearly half of the patients with IET, lesions were bilateral. Most extrathyroidal lesions were located directly below the right lobe. These observations are not fully consistent with previously published ones, because IET locations described by other authors varied. Bang et al. (11) found all 15 IET lesions in the inferior part of the thyroid lobe and IET was bilateral only in four patients. Similarly, all eight IET lesions described by Escobar et al. (6) were located in the inferior part of the thyroid, but strangely no subject had bilateral lesions. On the contrary, Kabaalioğlu et al. (4) reported a similar occurrence of IET in the middle and lower parts of the thyroid lobes. Interestingly, among 14 cases, only two IET cases were bilateral (4). Kim et al. (3) reported four IET cases in the middle part of the thyroid lobe while the remaining 8 IET cases were in the inferior part. Bilateral IET was observed in 3 cases (3). The predominance of IET occurrence in the middle part of the thyroid lobe was observed by Yildiz et al. (10), who reported 11 IET cases, among which 10 were located in the middle part and one in the inferior part of the lobe. Once again, none of the IET cases were bilateral (10). In two of 12 children with IET described by Frates et al. (12), the lesions were bilateral, and—interestingly—one of the lesions was located in the upper part of the thyroid. Comparing our observations and other authors' findings, it is clearly visible that IET is located in the middle or in the inferior part of the thyroid lobe, and extremely rarely it occurs in the upper part. However, it is a surprising issue that some of the authors did not observe the existence of bilateral IET (6, 10). Even when considering all our ET lesions together (extra-and intrathyroidal), the frequency of bilateral and unilateral ET occurrence is similar in our group. Perhaps this discrepancy is due to the fact that some very small lesions, located in the inferior pole of the thyroid lobes, were difficult to visualize and may have been neglected in other studies. The age does not seem to be decisive for the frequency of bilateral IET, as in our group the patients with bilateral lesions were 4–7 years old, similarly as in other authors' reports (4–9 years) (4), while in much younger children no bilateral IET cases were reported (11). It seems, therefore, that at least until the onset of puberty when the physiological process of thymic involution begins, the age does not affect the incidence and location of IET.

In US, most of our analyzed IET lesions were fusiform or ovular in shape, and one lesion had a very characteristic longitudinal shape which strongly suggested ectopic tissue rather than thyroid nodule. All the lesions were solid, hypoechoic, and heterogeneous with bright internal punctual or linear echoes. Generally, linear echoes are easier to differentiate from microcalcifications while punctual ones can look exactly like microcalcifications. It is known that both bright echoes in ET and microcalcifications in thyroid nodules are not evenly distributed within the lesions and can occur at one (in IET mainly central) or more parts of the lesion or can be scattered unevenly.

Most of ETs typically have well-defined margins, but sometimes the margins can be blurred or irregular, which can additionally suggest malignancy. Blood flow in PD is known to be decreased or absent. Our observations are similar to other authors' findings (3–6, 9–13). In our group, IET cases were hypovascular or no vascularity was visible on PD. Yildiz et al (9, 10) reported a group of patients in whom a few lesions were isovascular comparing to the thyroid parenchyma. Unfortunately, most authors did not report IET-related vascularity at all.

In our analyzed group of children, and in other described cohorts (5), the prevalence of IET was higher in males than females, although a few authors reported higher frequency in females (3). The mean age was also similar to those reported by other authors (3–6, 9–13). The largest dimension of ET lesion in or group was 4–18 mm, while other authors reported ET size from 3 to 33 mm (3, 4, 6, 11, 12). The largest ET dimensions were reported by Bang et al. (11), and in this group half of the patients were younger than 1 year old. In older children the size of ET was smaller but still very diverse (3–27 mm) and did not seem to depend on the age of the child at the time of diagnosis (3, 4, 6, 12). In all our cases, the ET tissue was similar in the US pattern to the normal descended thymus which was visible in every subject.

It is known that the IET tissue actually resembles the US pattern of papillary carcinoma and EET mimics metastatic lymph nodes. Distinguishing the suspicious thyroid nodule from IET in US requires a lot of experience. The lack of visible metastatic lymph nodes, which are typically present in children with PTC, cannot be used to exclude malignancy. Punctual bright internal echoes in ET are virtually impossible to differentiate from microcalcifications in US. In ET with linear bright echoes, such differentiation is possible, but only if a very experienced sonographer is available. However, in pediatric patients with hypoechoic thyroid nodule containing bright internal echoes resembling microcalcifications, the exclusion of malignancy cannot be based only on US examination. In our study, the usefulness of elastography in the differential diagnosis of IET and PTC has been demonstrated, but this method still does not provide a definitive diagnosis. Clinicians and radiologists should be aware of typical locations, US patterns and elastography features of ET, so as not to trigger unnecessary anxiety in the patient and his/her parents, and not to suggest the presence of PTC and unnecessarily refer to surgery. However, due to the high risk of cancer in thyroid nodules in children (1, 2), one should not determine the diagnosis only on the US image, neglecting further evaluation. It seems that such an approach can be recommended only when the US evaluation is done by a very experienced ultrasonographer, as there is a possibility of regular US monitoring, and—at the same time—there are contraindications to FNAB, resulting—for example—from contraindications to even short-term sedation in a very young child. One should always remember that ET and PTC may look similar. Moreover, it should be underlined that rare cases of thymoma, thymic carcinoma and lymphoblastic lymphoma arising from ET were reported (4, 15–18). Therefore, whenever possible, the diagnosis of cervical ET should be confirmed cytologically, although it may be difficult to aspirate diagnostic material from ET. Only cytological confirmation ensures that the evaluated lesion is a benign ET that requires simple periodical US monitoring. There are known cases when thyroidectomy was performed due to the lack of diagnostic material from FNAB in IET with suspicious US findings (19).

In conclusion, despite the low prevalence of IET and cervical EET, clinicians and radiologists should be aware of US characteristics of such lesions and of the necessity of confirmation of their benign character. Elastography is a useful tool to initially differentiate PTC and IET. However, due to the high risk of malignancy in thyroid lesions in children, similarity of US features of PTC and IET, and because of the possibility of malignancy in ET, only cytological evaluation provides definitive diagnosis and prevents, on one hand, unnecessary frequent diagnostic procedures and/or surgery and, on the other hand, missing malignant lesions.

## Ethics Statement

This study was carried out in accordance with the recommendations of WHO′ Standards and operational guidance for ethics review of health-related research with human participants, with written informed consent from all subjects' parents. All subjects' parents gave written informed consent in accordance with the Declaration of Helsinki. The protocol was approved by the Ethic Committee of Polish Mother's Memorial Hospital–Research Institute, Lodz, Poland.

## Author Contributions

MS was responsible for study design, data collection, data analysis, and writing of the manuscript. ZA, RS, and MT contributed to data collection and data analysis. TK contributed to data collection and was responsible for cytology smear assessment. AL contributed to study design, and writing of the manuscript. All authors were involved in writing the paper and approved the submitted final versions.

### Conflict of Interest Statement

The authors declare that the research was conducted in the absence of any commercial or financial relationships that could be construed as a potential conflict of interest.

## Abbreviations

EET: extrathyroidal ectopic thymic tissue
ET: ectopic thymic tissue
IET: intrathyroidal ectopic thymus
PD: power Doppler
PTC: papillary thyroid carcinoma
SR: strain ratio
US: ultrasound.

## References

[1] Francis GL Waguespack SG Bauer AJ Angelos P Benvenga S Cerutti JM American thyroid association guidelines task force. management guidelines for children with thyroid nodules and differentiated thyroid cancer. Thyroid. (2015) 25:716–59. 10.1089/thy.2014.0460 25900731 PMC4854274

[2] Niedziela M Handkiewicz-Junak D Małecka-Tendera E Czarniecka A Dedecjus M Lange D. Diagnostics and treatment of differentiated thyroid carcinoma in children - guidelines of polish national societies. Endokrynol Pol. (2016) 67:628–42. 10.5603/EP.2016.0072 28042655

[3] Kim HG Kim MJ Lee MJ. Sonographic appearance of intrathyroid ectopic thymus in children. J Clin Ultrasound. (2012) 40:266–71. 10.1002/jcu.21898 22362225

[4] Kabaalioğlu A Öztek MA Kesimal U Çeken K Durmaz E Apaydın A. Intrathyroidal ectopic thymus in children: a sonographic survey. Med Ultrason. (2017) 19:179–84. 10.11152/mu-913 28440352

[5] Fukushima T Suzuki S Ohira T Shimura H Midorikawa S Ohtsuru A Thyroid examination unit of the radiation medical center for the fukushima health management survey. prevalence of ectopic intrathyroidal thymus in japan: the fukushima health management survey. Thyroid. (2015) 25:534–7. 10.1089/thy.2014.0367 25778711

[6] Escobar FA Pantanowitz L Picarsic JL Craig FE Simons JP Viswanathan PA. Cytomorphology and sonographic features of ectopic thymic tissue diagnosed in paediatric FNA biopsies. Cytopathology. (2018) 29:241–6. 10.1111/cyt.12529 29577488

[7] Adamczewski Z Dedecjus M Skowrońska-Jóźwiak E Lewiński A Metastases of renal clear-cell carcinoma to the thyroid–a comparison of shear-wave and quasi-staticelastography. Pol Arch Med Wewn. (2014) 124:485–6. 10.20452/pamw.2413 24995474

[8] Ruchała M Szmyt K Sławek S Zybek A Szczepanek-Parulska E. Ultrasound sonoelastography in the evaluation of thyroiditis and autoimmune thyroid disease. Endokrynol Pol. (2014) 65:520–6. 10.5603/EP.2014.0071 25554621

[9] Yildiz AE Elhan AH Fitoz S. Prevalence and sonographic features of ectopic thyroidal thymus in children: a retrospective analysis. J Clin Ultrasound. (2018) 46:375–9. 10.1002/jcu.22590 29575022

[10] Yildiz AE Ceyhan K Sıklar Z Bilir P Yağmurlu EA Berberoğlu M. Intrathyroidal ectopic thymus in children: retrospective analysis of grayscale and doppler sonographic features. J Ultrasound Med. (2015) 34:1651–6. 10.7863/ultra.15.14.10041 26269296

[11] Bang MH Shin J Lee KS Kang MJ. Intrathyroidal ectopic thymus in children: a benign lesion. Medicine. (2018) 97:e0282. 10.1097/MD.0000000000010282 29620644 PMC5902273

[12] Frates MC Benson CB Dorfman DM Cibas ES Huang SA. Ectopic intrathyroidal thymic tissue mimicking thyroid nodules in children. J Ultrasound Med. (2018) 37:783–91. 10.1002/jum.14360 28850707

[13] Chng CL Kocjan G Kurzawinski TR Beale T. Intrathyroidal ectopic thymic tissue mimicking thyroid cancer in children. Endocr Pract. (2014) 20:e241–5. 10.4158/EP14236.CR 25148819

[14] Tian W Hao S Gao B Jiang Y Zhang X Zhang S. Comparing diagnostic accuracy of RTE and SWE in differentiating malignant thyroid nodules from benign ones: a meta-analysis. Cell Physiol Biochem. (2016) 39:2451–63. 10.1159/000452513 27832644

[15] Wu SL Gupta D Connelly J. Adult ectopic thymus adjacent to thyroid and parathyroid. Arch Pathol Lab Med. (2001) 125:842–3. 10.1043/0003-9985(2001)125<0842:AETATT>2.0.CO;2 11371249

[16] Büyükyavuz I Otçu S Karnak I Akçören Z Senocak ME. Ectopic thymic tissue as a rare and confusing entity. Eur J Pediatr Surg. (2002) 12:327–9. 10.1055/s-2002-35961 12469260

[17] Hirokawa M Miyauchi A Minato H Yokoyama S Kuma S Kojima M. Intrathyroidal epithelial thymoma/carcinoma showing thymus-like differentiation; comparison with thymic. APMIS. (2013) 121:523–30. 10.1111/apm.12017 23176314

[18] Pan XB Lang ZQ Cai L. Primary T lymphoblastic lymphoma arising from ectopic thymus in the neck of a child. Zhonghua Er Bi Yan Hou Tou Jing Wai Ke Za Zhi. (2011) 46:159–60. 21426717

[19] Durmaz E Barsal E Parlak M Gurer I Karaguzel G Akcurin S. Intrathyroidal ectopic thymic tissue may mimic thyroid cancer: a case report. J Pediatr Endocrinol Metab. (2012) 25:997–1000. 10.1515/jpem-2012-0207 23426832

