# Supplementary material for: Sonographic and Elastographic Features of Extra- and Intrathyroidal Ectopic Thymus Mimicking Malignancy: Differential Diagnosis in Children
Source: Front Endocrinol (Lausanne). 2019 Apr 10;10:223. doi: 10.3389/fendo.2019.00223 (PMC6499194; doi:10.3389/fendo.2019.00223)
Supplement: Supplementary file 1 [file Table_1.DOCX]

Supplementary Table 1. Clinical characteristics of the patients with ectopic thymic tissue

| Case | Age | Gender | TSH  (mIU/l) | FT4  (ng/dl) | FT3  (pg/ml) | aTPO  (IU/ml) | aTg  (IU/ml) | TRAb  (IU/ml) | PTH  (pg/ml) |
| --- | --- | --- | --- | --- | --- | --- | --- | --- | --- |
| 1. | 6 mo | F | 1.62 | 1.38 | NA | NA | NA | NA | 50.38 |
| 2. | 5 yr | M | 4.55 | 1.31 | 4.45 | 16.87 | < 10.0 | < 0.3 | 24.53 |
| 3. | 7 yr | M | 4.38 | 1.39 | 4.38 | 14.06 | 11.35 | < 0.3 | 31.18 |
| 4. | 4 yr | M | 3.66 | 1.44 | 4.62 | 9.84 | < 10.0 | < 0.3 | 23.97 |
| 5. | 10 yr | F | 1.95 | 1.07 | 3.89 | 23.5 | 11.7 | < 0.3 | 29.8 |
| 6. | 4 yr | M | 3.76 | 1.40 | 3.61 | 8.3 | < 10.0 | < 0.3 | 25.7 |
| 7. | 5 yr | F | 2.29 | 1.25 | 3.60 | 23.4 | 15.1 | 0.55 | 38.8 |
| 8. | 11 yr | M | 6.10 | 1.09 | 2.87 | 5.8 | 15.3 | < 0.3 | 22.5 |
| 9. | 6 yr | M | 4.51 | 1.32 | 3.02 | <5.0 | < 10.0 | < 0.3 | 35.7 |

Abbreviations: aTg, thyroglobulin antibodies; aTPO, thyroid peroxidase antibodies; ET, ectopic thymus; F, female; FT4, free thyroxine; FT3, free triiodothyronine; M, male; mo, months; NA, not available; PTH, parathyroid hormone;

TSH, thyroid stimulating hormone; TRAb, TSH receptor antibodies; yr, years.

Reference ranges: TSH 0.7-5.97 mIU/l for children < 6 yr, 0.6-4.48 mIU/l for children 6-12 yr; FT4 0.97-1.67 ng/dl; FT3 2.53-5.22 pg/ml; aTPO < 34 IU/ml; aTg < 115 IU/ml; TRAb < 1.75 IU/ml; PTH 15-65 pg/ml
